# Evolution of hepatitis B serological markers in HIV coinfected patients: a case study

**DOI:** 10.1590/S1518-8787.2017051006693

**Published:** 2017-03-31

**Authors:** ALCC Toscano, MC Mendes Corrêa

**Affiliations:** I Instituto de Infectologia Emílio Ribas. São Paulo, SP, Brasil; II Departamento de Doenças Infecciosas. Faculdade de Medicina. Universidade de São Paulo. São Paulo, SP, Brasil; III Instituto de Medicina Tropical de São Paulo. Laboratório de Investigação Médica 52. São Paulo, SP, Brasil

**Keywords:** HIV infection, Hepatitis B, Chronic, immunology, Coinfection, Biomarkers, Seroepidemiologic Studies

## Abstract

**OBJECTIVE:**

To describe the evolution of serological markers among HIV and hepatitis B coinfected patients, with emphasis on evaluating the reactivation or seroreversion of these markers.

**METHODS:**

The study population consisted of patients met in an AIDS Outpatient Clinic in São Paulo State, Brazil. We included in the analysis all HIV-infected and who underwent at least two positive hepatitis B surface antigen serological testing during clinical follow up, with tests taken six months apart. Patients were tested with commercial kits available for hepatitis B serological markers by microparticle enzyme immunoassay. Clinical variables were collected: age, sex, CD4+ T-cell count, HIV viral load, alanine aminotransferase level, exposure to antiretroviral drugs including lamivudine and/or tenofovir.

**RESULTS:**

Among 2,242 HIV positive patients, we identified 105 (4.7%) patients with chronic hepatitis B. Follow up time for these patients varied from six months to 20.5 years. All patients underwent antiretroviral therapy during follow-up. Among patients with chronic hepatitis B, 58% were hepatitis B “e” antigen positive at the first assessment. Clearance of hepatitis B surface antigen occurred in 15% (16/105) of patients with chronic hepatitis B, and 50% (8/16) of these patients presented subsequent reactivation or seroreversion of hepatitis B surface antigen. Among hepatitis B “e” antigen positive patients, 57% (35/61) presented clearance of this serologic marker. During clinical follow up, 28.5% (10/35) of those who initially cleared hepatitis B “e” antigen presented seroreversion or reactivation of this marker.

**CONCLUSIONS:**

Among HIV coinfected patients under antiretroviral therapy, changes of HBV serological markers were frequently observed. These results suggest that frequent monitoring of these serum markers should be recommended.

## INTRODUCTION

Among the estimated 40 million people living with human immunodeficiency virus (HIV) worldwide, it is believed that approximately four million (10%) are coinfected with hepatitis B virus (HBV)^1^. This is mainly because both viruses share the same routes of transmission.

The presence of HIV seems to alter the natural evolution of hepatitis B infection. It is believed that the presence of HIV could decrease the chance of HBV viral clearance after an acute infection and increase the risk for HBV chronic infection. Individuals with HIV-HBV coinfection have higher HBV DNA levels, rapid liver disease progression, and increased liver disease-related mortality, when compared to HBV monoinfected patients^2^.

Immunosuppression associated with HIV infection or interruption of anti-HBV treatment may lead to HBV reactivation, even in individuals with evidence of prior positive antibody to the hepatitis B surface antigen (anti-HBs)^3,4^. Furthermore, immunosuppression associated with HIV infection may be related to the inability to produce antibodies in the presence of an HBV infection. Therefore, atypical serological patterns have been described in this population. The presence of HBV variants with specific mutations has also been frequently described in the same population, and has been associated with some atypical serological patterns found in coinfected patients^5^.

Evolution of hepatitis B serological markers among HIV coinfected patients has been evaluated by some authors, and these studies reveal great variation of these markers during clinical follow up^6,7^. In Brazil, however, data regarding this aspect of HBV infection in this population is scarce.

This study aimed to describe the evolution of hepatitis B surface antigen (HBsAg) and hepatitis B “e” antigen (HBeAg) among HIV coinfected patients, with emphasis on the reactivation or seroreversion of these markers in this group of patients.

## METHODS

The enrolled patients were selected from those regularly followed up at an HIV Outpatient Clinic in São Paulo, Brazil, between May 2006 and July 2011. All HIV-infected patients with serum hepatitis B surface antigen HBsAg were identified. HBsAg-positive patients who underwent at least two repeated HBV serological testing, during clinical follow up, with tests taken six months apart, were included in the analysis. The study was conducted from June 2011 to July 2012.

The search for information about HBsAg-reactive patients started with an electronic and written database query. The serological profile analysis relative to hepatitis B in all patients with regular treatments during the determined time frame (between May 2006 and July 2011) identified HBsAg positive patients. We included all serological tests found for each patient, containing serological markers of HBV infection.

Medical records were reviewed retrospectively to ascertain demographic and clinical characteristics and laboratory findings during the six months preceding the serological test of interest (we considered the data sample in which serological reactivation of HBsAg and/or HBeAg markers was first detected).

The following clinical and demographic variables were considered for analysis: sex, age, HIV or HBV risk factors, treatment adherence; CD4+ T-cell count; HIV viral load, alanine aminotransferase (ALT) level; use and duration of exposure to antiretroviral drugs (ARV); use and duration of use of HBV-active drugs (lamivudine, tenofovir, entecavir, or interferon). Epidemiological, clinical, and laboratory features were described based on the date of serological test of interest.

For each patient, we calculated the mean ALT level and CD4 count measurement for all measurements performed six months prior to each HBV serological test. HIV viral load was considered detectable when present in any sample collected during the six months before the serological test of interest. The absolute values of HIV viral load will not be described, since the long period of follow-up did not allow the analysis using a single method.

For the purpose of this study, we considered HBsAg or HBeAg seroclearance when HBsAg or HBeAg, respectively, became non-reagent after being reagent, during clinical follow up.

HBsAg or HBeAg seroreversion was considered whenever these markers were detected reagent after being previously detected as non-reagent (with no appearance of anti-HBs or anti-HBe, respectively) during clinical follow up.

HBsAg or HBeAg reactivation were considered whenever these markers were detected reagent after been previously detected as non-reagent (with appearance of anti-HBs or anti-HBe, respectively) during clinical follow-up. Follow up time was considered as the period of time between the first and last serologic sample containing HBsAg or HBeAg markers.

HIV results were obtained using two commercial enzyme immunosorbent assays (Organon Technika, Tournault, Belgium and Embrabio, São Paulo, Brazil) and confirmed using GS-HIV-1 Western Blot (Bio-Rad, Hercules, CA). HIV RNA was quantified using the Versant HIV-1 RNA 3.0 bDNA assay (Siemens Healthcare, Erlangen, Germany) and Nucleic Acid Sequence-Based-Amplification (NASBA-NUCLISENS). CD4 T-lymphocyte counts were determined using the BD Multitest/Trucount (BD Biosciences, San Jose, CA).

A microparticle enzyme immunoassay (MEIA, AxSYM, Abbott Laboratories, Abbott Park, IL) was used to detect serological markers (HBsAg, anti-Hepatitis B core total antibodies, anti-HBs, HBeAg, and anti-HBe) at the Central Laboratory of the Hospital das Clínicas, São Paulo, Southeastern Brazil.

This project was approved by the Research Ethics Committee of the Department of Infectious and Parasitic Diseases of the Faculdade de Medicina da Universidade de São Paulo (CAPPesq Protocol 0197/11, from November 5, 2011). It was exempted of the need for an informed consent form for patients. Secrecy and confidentiality were guaranteed in this study.

The analyses were performed from a database entered into Microsoft Excel^®^. A descriptive analysis of the study variables was carried out.

Due to the small number of patients included in the study, we were not able to compare the possible associations between the different patterns of serological evolution observed and the clinical or epidemiological variables described.

## RESULTS

Initially, 2,242 patients were enrolled in the AIDS Outpatient Clinic with confirmed HIV infection and serological markers for hepatitis B. Of those, 105 (4.7%) were patients with chronic hepatitis B*.*

The clinical and epidemiological features of the 105 included HBsAg-positive patients are shown in Table 1.

**Table 1:** Epidemiological, clinical, and laboratory features of 105 HBsAg-reactive patients coinfected with HIV.

Demographic and epidemiological variables	n	%
Men	102	97.1
Mean age (years)	50.4	SD = 8.2
Background MSM	54	51.4
Background IDU	6	5.7
Clinical variables		
Previous ARV use	105	100
Previous lamivudine use	101	96.0
Previous tenofovir use (TDF)	78	74.0
Previous LAM, TDF, ENT, or IFN use	101	96.0
Previous entecavir use (ENT)	4	3.8
Previous interferon use (IFN)	5	4.7
History of AIDS-defining illness	46	43.8
Laboratory variables		
HBeAg reactivity	61	58.0
Mean CD4+ T-cells	4,492	Variation: 10-1,536 SD = 211.5 cells/mm^3^
Elevation ALT >1.5 × ULN^†^	61	58.0

MSM: men who have sex with men; IDU: injection drug use; ARV: antiretroviral drug; TDF: Tenofovir; LAM: Lamivudine; ENT: Entecavir; IFN: Interferon; ALT: alanine aminotransferase; ULN: upper limit of normality

Most patients were male (97%), with the main risk factor for exposure to both infections (HIV and HBV) being risky sexual behavior (including same-sex relations). All patients underwent antiretroviral therapy during the monitoring phase. A total of 44% (46/105) of patients had a history of one or more previous AIDS defining illness. CD4+ T-cell counts varied from 10 to 1,536 cells/mm^3^, with the average count being 449.2 cells/mm^3^(standard deviation [SD] = 211.5 cells/mm^3^). A total of 101 (96%) and 78 (74%) patients used lamivudine or tenofovir, respectively, in their therapeutic regimen during follow up.

Follow up time for these 105 patients varied from six months to 20.5 years.

The number of serological testing for each patient varied from two to 18 along this period.

Among those with HBsAg positive, 58% (61/105) were also HBeAg positive.

Clearance of HBsAg occurred in 15% (16/105) of patients with chronic hepatitis B (1.7 cases per 100 person-years), and 50% of them (8/16) presented subsequent seroreversion or reactivation of HBsAg during clinical follow up. Two patients (25%) presented anti-HBs before HBsAg reactivation.

Follow up time for patients who underwent HBsAg reappearance varied from 1,775 to 6,051 days (4.9 years to 16.8 years).

Clinical characteristics of patients who presented HBsAg reactivation are summarized in Table 2.

**Table 2:** Clinical characteristics of patients who underwent HBsAg reappearance.

ID	HBeAg at first assessment	Mean CD4 count at seroreversion detection	History of AIDS-defining illness	ARV exposure	Detectable HIV viremia at seroreversion detection	Exposure to anti-hepatitis B drugs before seroreversion detection	History of anti-hepatitis drugs interruption before seroreversion detection	ALT level at seroreversion detection
1	Non reagent	380	No	Yes	No	No	NA	1.5 to 2.5 ULN
2	Non reagent	462	No	No	Yes	No	NA	< 1.5 ULN
3	Reagent	448	No	Yes	Yes	LAM	No	2.5 to 3.5 ULN
4	Reagent	618	No	Yes	Yes	No	NA	3.5 to 5 ULN
5	Non reagent	546	No	Yes	No	LAM	No	< 1.5 ULN
6	Reagent	422	No	Yes	No	No	NA	< 1.5 ULN
7	Non reagent	478	Kaposi Sarcoma	Yes	Yes	LAM	No	< 1.5 ULN
8	Non reagent	365	No	Yes	No	LAM+TDF	No	< 1.5 ULN

HBsAg: hepatitis B surface antigen; HBeAg: hepatitis B “e” antigen; ARV: antiretroviral drug; LAM: Lamivudine; TDF: Tenofovir; NA: not applicable; ALT: alanine aminotransferase; ULN: upper limit of normality

Among HBeAg positive patients at the first serologic test, 57% (35/61) underwent clearance of this serologic marker (5.8 cases per 100 person-years). During clinical follow up, 28.5% (10/35) of those who initially cleared HBeAg presented HBeAg reactivation or seroreversion. Five patients (50%) presented anti-HBe before HBeAg reactivation.

Follow up time for patients who underwent HBeAg reappearance varied from 1,296 to 5,087 days (3.6 years to 14.1years).

Clinical characteristics of these patients are described in Table 3. All patients were male, with average age of 56 years (SD = 11). The average CD4+ T-cell count was 588 cells/mm^3^.

**Table 3:** Clinical characteristics of patients who underwent HBeAg reappearance.

ID	HBeAg at first assessment	Mean CD4 count at seroreversion detection	History of AIDS-defining illness	ARV exposure	Detectable HIV viremia at seroreversion detection	Exposure to anti hepatitis B drugs before seroreversion detection	History of anti-hepatitis drugs interruption before seroreversion detection	ALT level at seroreversion detection
10	reagent	682	No	No	Yes	No	NA	< 1.5 ULN
17	reagent	871	No	Yes	Yes	LAM+TDF	Yes	1.5 to 2.5 ULN
18	reagent	613	No	Yes	No	LAM	No	< 1.5 ULN
19	reagent	494	No	Yes	Yes	LAM+TDF	Yes	< 1.5 ULN
20	reagent	431	No	Yes	No	LAM	No	1.5 to 2.5 ULN
21	reagent	292	Lung Tb	No	No	No	NA	> 5 ULN
22	reagent	498	No	Yes	No	LAM	No	1.5 to 3.5 ULN
23	reagent	739	No	Yes	No	LAM+TDF	No	1.5 to 2.5 ULN
24	reagent	858	No	Yes	No	LAM+TDF	No	< 1.5 ULN
25	reagent	402	No	No	Yes	No	NA	< 1.5 ULN

HBeAg: hepatitis B “e” antigen; Tb: tuberculosis; ARV: antiretroviral drug; LAM: Lamivudine; TDF: Tenofovir; NA: non aplicable; ALT: alanine aminotransferase; ULN: upper limit of normality

## DISCUSSION

In this study, changes of HBV serological markers were frequent among HIV-HBV coinfected patients during their clinical follow up. We identified high initial HBsAg and HBeAg clearance rates, at 15.2% and 57%, respectively. Then, we observed HBsAg and HBeAg reappearance in 50% and 28.6% of patients, respectively, throughout their clinical monitoring.

HBsAg and HBeAg serological clearance has been analyzed and extensively described among monoinfected HBV patients. This is a phenomenon observed in the natural course of this infection and depends on different factors. During hepatitis B treatment, the type of drug and period of exposure to a specific regimen are associated with different clearance rates observed throughout clinical monitoring for both antigens; however, the clearance rates increase with monitoring time^7,8^.

Few studies have evaluated this issue among HIV coinfected patients, yet our study confirms what was previously evaluated by some authors. After five years of monitoring, Sheng et al.^9^found an HBsAg clearance rate of 14.4% in HIV coinfected patients. On the other hand, Nunez et al.^10^ and Maylin et al.^11^ observed a loss of HBsAg and HBeAg, varying from 2.8 to 13% and 17.7 to 27.7%, respectively.

Overall, the adequate use of ARV may have an important impact on the natural course of HIV infection, restoring specific adaptive and nonspecific innate immune responses^2,12^. It is important to emphasize that this impact is independent of the use of drugs with anti-HBV action in this medication regimen. The use of lamivudine, emtricitabine, or tenofovir enhances this impact, as HBV infection replication decreases, thus contributing concurrently and significantly to the patient’s overall immune response restoration. Coinfected patients with no treatment exhibit a lower average CD4+ count and greater HIV viral loads when compared to HBV monoinfected individuals^13,14^.

Therefore, it is possible to suppose that the high HBsAg and HBeAg clearance rates observed among coinfected patients may be partly due not only to the antiviral action of anti-HBV drugs used in most patients, but also to the immunomodulatory action of the medication therapy as a whole. In accordance to this, HBsAg seroconversion to anti-HB has been frequently described after introduction of antiretroviral treatment in this population^15^.

High rates of HBsAg and HBeAg reappearance were observed in our study. Matthews et al.^16^ identified HBsAg and HBeAg reactivation, respectively, in one and two patients, among the 47 monitored over 42 months. Di Martino et al.^17^ observed HBeAg reversion in five patients, among the 14 monitored over 24 months, who were subjected to interferon therapy for chronic hepatitis B treatment.

It is possible that different causes might be associated with the frequent serological variations observed in our study. First, to the best of our knowledge, until now, no other research group has monitored patients’ HBV serological markers, among HIV infected patients using ARV, for such a long time. Second, aspects related to the sensitivity of the serological methods involved could also be associated with some of the serological alterations observed among our patients. Small titer oscillations of those markers above or below the detection limit could eventually have generated some of the alterations observed in the patients included in our study; as such, the HBsAg or HBeAg reemergence may not have represented real reactivations, but instead titer oscillations of these markers. It is also possible that intercurrent plasmatic factors associated with other concomitant infections in this group of patients could have, in some way, interfered with some of the results observed. In this sense, Rabenau et al.^18^, using the AxSYM^TM^ method for HBsAg detection, described significant statistical differences between samples of anti-HB-positive and anti-HB-negative plasma saturated with HBsAg.

Third, it is possible to suppose that some of the included patients could have selected mutations in the surface antigen genes or in the precore/core genes. The presence of these mutations could have been associated with the absence of HBsAg or HBeAg in some of the included patients. In this regard, a recent study evaluated alterations in the HBV genome among coinfected patients over time, and identified important evolutionary alterations in the patients’ viral genome^19^. Numerous amino acid variations were found; however, the mutants specifically selected for defects in diagnostic tests were not observed in that particular study.

Also, all patients included in this study were under ARV. Among them, 96% had used lamivudine and 74% had used tenofovir, according to medical records. Therefore, nearly 26% of patients were submitted to lamivudine monotherapy for some time. Lamivudine monotherapy is frequently associated with the selection of mutants in the polymerase region^20^. In this case, mutations in the S gene region could eventually have been selected among the analyzed patients^21^. Unfortunately, HBV DNA quantification or the identification of mutations in the serum of these patients was not available for analyses.

The presence of the virus genotype G among the patients could also have contributed to the HBeAg variations observed. Genotype G has been associated with the presence of two codons responsible for the translation termination in the HBV precore region, which seem to inhibit the synthesis of HBeAg^22^. It is interesting to note that the presence of genotype G was previously described by other authors in a few patients from the same study population^23^. The presence of the HBV genotypes C and F could also have been associated with a greater chance of HBeAg seroreversion in that population^24^.

It is important to mention, however, that this study has some limitations. It was a retrospective analysis of the medical records, which makes it impossible to precisely evaluate some aspects regarding patients’ general adherence to treatment. Similarly, the lack of prospective and systematically collected ALT data may have limited our observation of ALT alterations preceding the episodes of reemergence of HBsAg or HBeAg. However, we emphasize that although there was no acute evidence of ALT elevations in the group of patients analyzed, the impact of HBsAg and HBeAg marker reactivation or seroreversion, in increased disease severity, should not be discarded. Studies on HBV monoinfection have clearly associated HBsAg reactivation and HBeAg seroreversion with a worse prognosis of liver disease^25^. Also, the small sample of patients included did not allow us to analyze possible associations between the evolution of serological markers and the clinical and laboratory variables described.

In conclusion, changes of HBV serological markers were frequently observed in our study. High HBsAg and HBeAg clearance rates, as well as reemergence of these markers, were also observed in this population. Our data suggest that periodic measurements of HBV serological markers should be recommended.
